# Anterior pituitary hormones in first-episode psychosis: a systematic review and meta-analysis

**DOI:** 10.1192/j.eurpsy.2023.588

**Published:** 2023-07-19

**Authors:** D. Cavaleri, C. A. Capogrosso, P. Guzzi, B. Misiak, G. Bernasconi, M. Re, C. Crocamo, F. Bartoli, G. Carrà

**Affiliations:** ^1^Department of Medicine and Surgery, University of Milano-Bicocca, Monza, Italy; ^2^Department of Psychiatry, Wroclaw Medical University, Wroclaw, Poland; ^3^Division of Psychiatry, University College London, London, United Kingdom

## Abstract

**Introduction:**

Although the role of pituitary gland in schizophrenia and psychotic disorders has been studied for decades, evidence on anterior pituitary hormones in the early phases of psychoses – without the influence of chronicity, comorbidities, and pharmacological treatment – is mostly unclear and inconsistent.

**Objectives:**

Our systematic review and meta-analysis was aimed at comparing the blood concentrations of adrenocorticotropic hormone (ACTH), follicle stimulating and luteinizing hormones (FSH and LH), growth hormone (GH), prolactin (PRL), and thyroid-stimulating hormone (TSH) between people with drug-naïve first-episode psychosis (FEP) and healthy controls.

**Methods:**

We searched main electronic databases for articles indexed up to September 2022. We appraised the quality of data. We carried out random-effects meta-analyses, generating pooled standardized mean differences (SMDs) and estimating between-study heterogeneity. Moreover, we performed sensitivity and meta-regression analyses.

**Results:**

Twenty-six studies were included. People with drug-naïve FEP had higher ACTH (p<0.001; moderate-to-high heterogeneity) and PRL (p<0.001; high heterogeneity) concentrations, as well as lower TSH concentrations (p=0.001; low heterogeneity), than healthy subjects. Sensitivity analyses confirmed these findings. Data were not sufficient to perform meta-analyses on other hormones (FSH, LH, and GH).

**Conclusions:**

People with drug-naïve FEP have abnormal ACTH, PRL, and TSH blood concentrations, supporting the hypothesis that anterior pituitary hormone secretion is altered in the first stages of schizophrenia and psychoses. Additional research is needed to clarify the complex interconnections between vulnerability, environmental factors, and pituitary hormones in FEP.

**Disclosure of Interest:**

None Declared

